# Out of Sight, out of Mind? An Audit Which Proposes a Follow-Up and Management Pathway for Inferior Vena Cava Filters

**DOI:** 10.1155/2016/6538456

**Published:** 2016-03-27

**Authors:** Caitriona Logan, Niamh O'Connell, John Kavanagh, Niall McEniff, Mark Ryan, Michael Guiney, Orla Seery, James O'Donnell, Kevin Ryan, Barry White

**Affiliations:** National Center for Hereditary Coagulation Disorders, St. James's Hospital, Dublin 8, Ireland

## Abstract

Insertion of an IVC filter can be a safe and effective way to avoid PE in thrombosis patients who cannot be anticoagulated. If temporary filters are not promptly removed they can become difficult to remove, causing avoidable complications and often requiring lifelong warfarin. In this study, two sequential audits of retrieval of temporary IVC filters were conducted before and after the implementation of a coordinated management strategy for IVC filter follow-up. 33 filter placements were examined over a 15-month period (Group A). Following implementation of the strategy a comparable 15-month period in which 33 IVC filters were placed was audited (Group B). Following implementation, failed retrievals dropped from 15% to 9%. The number successfully retrieved did not change at 45%. The number made permanent from the outset following expert discussion increased from 12% to 39%. The number of filters with no attempted retrieval and no consultation about retrieval decreased from 27% to 9% (these patients were lost to follow-up with multiple contact attempts made). In Group B 100% of placed IVC filters were followed up appropriately. The proposed model is an easily implemented plan to avoid patient morbidity caused by temporary IVC filters made unintentionally permanent by loss to follow-up.

## 1. Introduction

Venous thromboembolism (VTE) encompassing deep vein thrombosis (DVT) and pulmonary embolus (PE) is a significant cause of morbidity and mortality worldwide. In a study of over 42 million deaths in the USA over a 20-year period pulmonary embolism was listed as a diagnosis in 1.5% and was the principal cause of death in 1/3 of these [1]. The mainstay of therapy to avoid preventable deaths by recurrent PE is parenteral and/or oral anticoagulants. However, contraindications to systemic anticoagulation may arise including active bleeding, thrombocytopenia, or planned imminent surgery [2, 3]. In these scenarios, interruption of the inferior vena cava (IVC) may be considered to prevent distal emboli from reaching the lungs [2]. Temporary IVC filter placement is a safe and effective preventative measure to avoid potentially life threatening pulmonary embolism [4–6]. There are some indications for IVC filter placement such as absolute contraindication to or failure of anticoagulation in the setting of VTE [3, 7]. Other indications are less definite and are subject to expert clinical judgement.

Modern filters are optimally retrieved within 14 days of placement but reportedly can be retrieved successfully up to 26 weeks. However, there are barriers to successful retrieval which result in published retrieval rates of only 20–40% in eligible patients [8]. Lack of appropriate follow-up, poor documentation of a retrieval plan, and cancer diagnosis have been indicated as reasons for such low retrieval rates [9, 10].

Patients with removal IVC filters require planned follow-up for several reasons. The window of retrievability may vary depending on the device chosen and this should be expressed in the radiological procedure report. Patient factors or the clinical scenario may change meaning that filter removal is no longer safe or no longer desired. Unfortunately, even when prompt retrieval is indicated, many filters are not actually removed with most studies showing removal rates of between 20 and 40% [8].

## 2. Methods

St. James's Hospital is a large, tertiary referral hospital, which has a National Cancer Centre for medical and surgical oncology as well as Ireland's National Comprehensive Care Center for Coagulation Disorders. In this study, two sequential audits of retrieval of temporary IVC filters were conducted before and after the implementation of a coordinated management strategy for IVC filter follow-up. The Royal College of Radiology (RCR) audit template from 2011 entitled “Attempted Retrieval of Temporary IVC Filters” [11] was used as an audit framework. The standard states that 100% of patients with a retrievable filter should have an attempt at retrieval (assuming the clinical circumstances do not change to require a permanent filter).

Data was retrieved from IR records, the hospital electronic patient record, and the paper medical records. The parameters examined were date of temporary IVC filter insertion, indication for insertion, type of filter and removal plan documentation, and date of attempted removal.

The initial audit cycle took place from 1 January 2012 until 31 March 2013, a 15-month period (Group A). Inclusion criteria for the control group, Group A, were any patients who had an IVC filter placed in our center from 1 January 2012 until 31 March 2013 for any indication.

Following the Group A audit, a multidisciplinary team involving the Interventional Radiology and Hematology Departments developed a framework for IVC filter follow-up. A three-point plan was devised to include the following:

1. A specialist nurse who led the register of all filters placed.
2. A coagulation hematology consult and clinic service for filters that may be required to be left in situ or complex removal decisions.
3. Beds in a day-ward that were made available for retrieval at short notice.

A seven-month window was given to fully implement the three-step plan. A comparable 15-month period was then audited (Group B). Included in Group B were all filters placed in our center between November 2013 and December 2014.

Groups A and B patients were only similar population groups in that they had a contraindication to parenteral anticoagulation and they had an IVC filter successfully placed (Table 1).

## 3. Results

**Figure 1:**
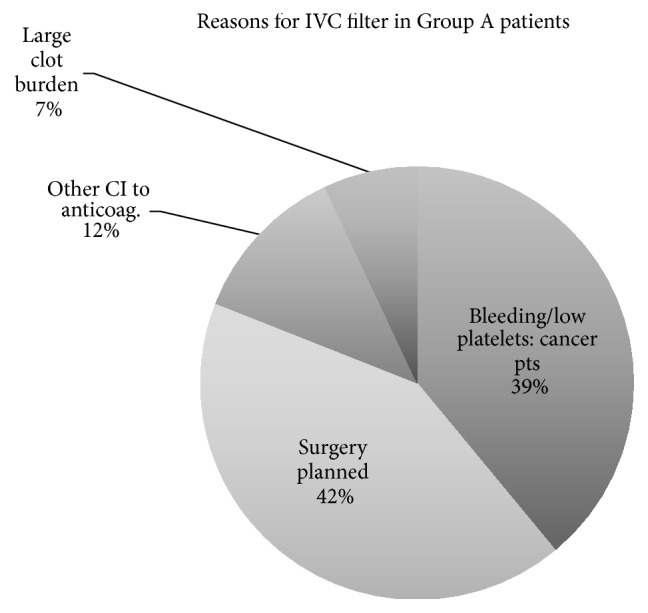
Indications for referral for IVC filters in Group A.

The control group included 33 patients (Group A). The indications for IVC filter placement are demonstrated in Figure 1.

**Figure 2:**
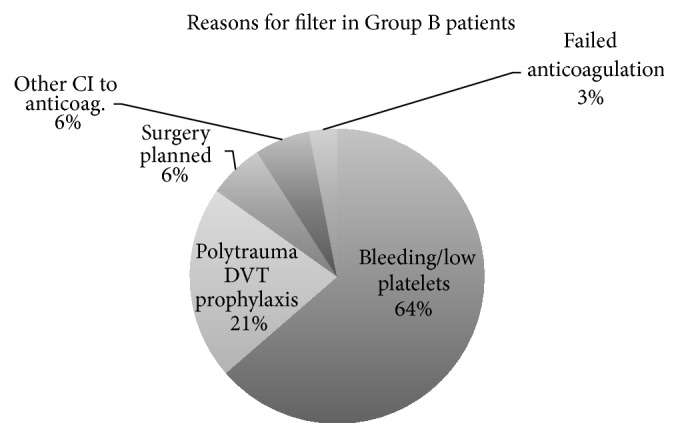
Indications for referral for IVC filters in Group B.

Reasons for filter placement in thrombosis patients in Group B are depicted in Figure 2.

29 of the 33 patients in Group A were eligible at least initially for filter removal with an average age of 62 years. Four were either made permanent at placement or the patients died (12%). Filter removal was scheduled and attempted in 72% of those eligible. In the 29 patients eligible for removal 15 (45%) had their filters removed successfully with mean number of days to successful retrieval 61. The longest time to successful retrieval was 230 days. Removal was attempted but unsuccessful in 5 patients 15% of the total, filter tilt and embedded struts being the main reasons why. The mean number of days to failed retrieval was 94 days. In 9 patients (31% of the patients eligible for retrieval) no retrieval was attempted and no reason was given as to why.

84% of the patients had documentation by the interventional radiologist either in the medical notes or the procedure report giving a timeframe in which removal should be considered. Approximately 70% of these alluded to success rates of future retrieval in certain timeframes.

Only 33% of the patients had documentation by the team about a removal plan even though many of those who did not still had their filter removed.

In Group B following implementation of the three-step plan 45% of the filters were removed successfully with the mean days to successful removal of 46. 9% had failed removal with mean days to unsuccessful retrieval of 92. Filter embedding into the IVC wall was the reason for failure in these patients. In 46% (*N* = 15) of patients the filters were not removed. 13 of these patients were seen by a hematology consultant and a decision was reached to leave the filter in permanently. In 3 patients (9%) no retrieval was attempted. In these cases multiple attempts were made to schedule filter consultations to which the patient did not attend.

91% of Group B had appropriate documentation by the interventional radiologist in either the medical notes or the procedure report giving a timeframe in which removal should be considered.

## 4. Discussion

In this study, following implementation of the IVC filter management plan, all patients who had temporary IVC filters were identified and offered follow-up if the filter remained in situ after six weeks (100% follow-up). Three patients were offered follow-up but did not attend the clinic visit despite repeated attempts of contact. In patients eligible for retrieval, 91% of patients had an attempted retrieval. Although this does not meet the RCR target of 100%, the patients in whom retrieval was not attempted were those who declined to attend.

There was a reduction of failed retrievals from 23% to 9% between the two groups. The time to retrieval was reduced from 62 days to 45 days. Documentation by the interventional radiologist to include advice about filter retrieval improved from 84% in Group A to 91% in Group B. The limitation of our study is its small size meaning the results do not reach statistical significance. The audit cycle does provide however a practical and easily implementable framework for filter follow-up which is relevant to any center routinely placing these devices.

## Figures and Tables

**Table 1:** Comparison of Groups A (control group) and B (following implementation of the three-step plan).

Group	Made permanent	Removed	Failed removal	No removal attempted
Group A (*N* = 33)	4 (12%)	15 (45%)	5 (15%)	9 (27%)
Group B (*N* = 33)	13 (39%)	15 (45%)	3 (9%)	3 (9%)
